# Antagonism of CD11b with Neutrophil Inhibitory Factor (NIF) Inhibits Vascular Lesions in Diabetic Retinopathy

**DOI:** 10.1371/journal.pone.0078405

**Published:** 2013-10-21

**Authors:** Alexander A. Veenstra, Jie Tang, Timothy S. Kern

**Affiliations:** 1 Department of Pharmacology, Case Western Reserve University, Cleveland, Ohio, United States of America; 2 Department of Medicine, Case Western Reserve University, Cleveland, Ohio, United States of America; 3 Stokes Veterans Administration Medical Center, Cleveland, Ohio, United States of America; Institut de la Vision, France

## Abstract

Leukocytes and proteins that govern leukocyte adhesion to endothelial cells play a causal role in retinal abnormalities characteristic of the early stages of diabetic retinopathy, including diabetes-induced degeneration of retinal capillaries. Leukocyte integrin αmβ2 (CD11b/CD18, MAC1), a protein mediating adhesion, has been shown to mediate damage to endothelial cells by activated leukocytes in vitro. We hypothesized that Neutrophil Inhibitory Factor (NIF), a selective antagonist of integrin αmβ2, would inhibit the diabetes-induced degeneration of retinal capillaries by inhibiting the excessive interaction between leukocytes and retinal endothelial cells in diabetes. Wild type animals and transgenic animals expressing NIF were made diabetic with streptozotocin and assessed for diabetes-induced retinal vascular abnormalities and leukocyte activation. To assess if the leukocyte blocking therapy compromised the immune system, animals were challenged with bacteria. Retinal superoxide production, leukostasis and leukocyte superoxide production were increased in wild type mice diabetic for 10 weeks, as was the ability of leukocytes isolated from diabetic animals to kill retinal endothelial cells in vitro. Retinal capillary degeneration was significantly increased in wild type mice diabetic 40 weeks. In contrast, mice expressing NIF did not develop any of these abnormalities, with the exception that non-diabetic and diabetic mice expressing NIF generated greater amounts of superoxide than did similar mice not expressing NIF. Importantly, NIF did not significantly impair the ability of mice to clear an opportunistic bacterial challenge, suggesting that NIF did not compromise immune surveillance. We conclude that antagonism of CD11b (integrin αmβ2) by NIF is sufficient to inhibit early stages of diabetic retinopathy, while not compromising the basic immune response.

## Introduction

Diabetic retinopathy (DR) is a leading cause of blindness in working age adults [1–3]. Vascular abnormalities in the retina, including capillary non-perfusion and degeneration, and vascular leakage are detectable in the early non-proliferative stages of the retinopathy. The progressive loss of retinal blood vessels and increased vascular leakage in the non-proliferative phase of DR is believed to cause ischemia and edema, respectively, which lead to progression of the retinopathy into the clinically significant, proliferative (neovascular) phase [4]. Improved glycemic control has been shown to inhibit the diabetes-induced loss of retinal capillaries in animals [5] and the progression of DR [6,7], however good glycemic control has been difficult to achieve, and therefore new therapeutic approaches are needed.

Also detectible in the early stages of diabetic retinopathy in both diabetic animals [8] and patients [9] is an increase in the number of leukocytes adhering to retinal blood vessels (leukostasis), and an increase in the expression of retinal intercellular adhesion molecule-1 (ICAM1, CD54). Leukocytes have been implicated in the loss of retinal capillaries, but the exact mechanism for this degeneration has remained elusive [10]. Leukocytes have been shown to occlude retinal capillaries in diabetic animals [8,11]. Furthermore some but not all of the occluded capillaries remain occluded by unknown mechanisms after the leukocytes leave the capillary demonstrating that temporary occlusion by leukocytes can lead to permanent occlusion and eventual capillary death[11,12]. More direct evidence for leukocyte mediated capillary loss has been demonstrated in long term diabetic mice in which only bone marrow derived cells, leukocytes, were genetically altered [13].

Leukocyte functions, such as adhesion, activation, priming, and respiratory burst are mediated in part by integrins in the CD11/CD18 family. CD11b/CD18 (α_m_β2, MAC-1), an integrin on the surface of leukocytes that binds many ligands including ICAM1 [14], has been shown to mediate in vitro endothelial damage and death caused by activated leukocytes [15]. In vivo antibody therapy against ICAM1 or CD18 has been reported to inhibit diabetes-induced retinal leukostasis and endothelial injury in mice, suggesting that integrin/CAMs might make a good target for therapy [11,16,17]. Additional anti inflammatory therapies have been reported which decrease diabetes-induced retinal leukostasis and permeability or retinal ICAM1 such as corticosteroids[18], RAGE inhibitors [19], and salicylates [20].

NIF is a selective antagonist of the α_m_β_2_ integrin (CD11b/CD18 or Mac1) but not other CD18 containing integrins such as CD11a/CD18 LFA-1 [21,22]. NIF blocks the binding of several α_m_β2 ligands, including ICAM-1, fibronectin, C3bi, and denatured protein, by blocking the pocket where the ligands bind, although the residues to which NIF binds on CD11b are different from the residues which bind other ligands [23,24]. Thus, NIF-mediated inhibition of the interaction between leukocytes and endothelial cells may offer advantages not seen with other approaches.

We present evidence that antagonism of the α_m_β_2_ integrin by NIF inhibits diabetes-induced degeneration of retinal capillaries, as well as inhibiting other abnormalities of retina that are believed to contribute to the vascular degeneration. Importantly, NIF did not impair the ability of leukocytes to resolve opportunistic bacterial infections. These findings suggest that the α_m_β_2_ integrin on leukocytes plays a critical role in diabetes-induced leukostasis, retinal production of superoxide, and degeneration of retinal capillaries, and that these abnormalities can be inhibited by NIF.

## Methods

### Ethics Statement

All experiments complied with guidelines established by The Association for Research in Vision and Ophthalmology (ARVO) and Case Western Reserve University (the Guide for the Care and Use of Laboratory Animals of the National Institutes of Health). The protocol was approved by the Institutional Animal Care and Use Committee of Case Western Reserve University (Protocol 2010-0156). Mice were terminally anesthetized with CO2 immediately prior to harvest of blood and tissues. Anesthesia for non-terminal procedures to reduce discomfort consisted of 0.1mL/20g body weight IP of 15mg Ketamine, 3mg Xylazine, 0.5mg Acepromazine / 1.4mL saline.

### Animals

Male offspring from NIF^+/-^ CD1 males [25] bred with female wild type (WT) mice (CD1, NIF^-/-^, Charles River) were separated by genotype NIF^+/-^ or WT and housed in filtered air cages. Diabetes was induced at approximately eight weeks of age in male mice fasted for 6 hours prior to daily IP injection of 200uL of sterile filtered (0.22um) streptozotocin (60 mg/kg ms) (MP Biomedical) in Citrate buffer (0.53mM Citric acid, 0.47mM Sodium Citrate pH 4.5) over the course of 5 days. Food was provided immediately after injection. The streptozotocin solution was used immediately after dissolving the stock powder in the citrate buffer (the solution is photosensitive and quickly self inactivates). Fasted blood glucose (6 hr fast) was obtained from tail blood at 7, 14 and 20 days after the last injection using EasyMax N blood glucose test strips and meter (Gemco medical, Ohio). Animals with fasted blood glucose greater than 250 mg/dL were assigned to the diabetic group. Diabetic mice were weighed weekly and body weight was maintained as needed (2g / 2 week loss threshold) with 0.1-0.2 units of Humulin N (NPH) insulin diluted 1:10 in sterile diluent (Eli Lilly) 2-3 times per week. Hyperglycemia was quantified via blood glucose concentrations, and every 2–3 months by glycated Hemoglobin A1c (Variant HbA1c; BioRad) [20,26,27]. Mice were harvested at 10 weeks and at 40 weeks duration of diabetes to evaluate retinal physiologic abnormalities and histopathology (capillary degeneration), respectively. NIF expressing mice [25] were the generous gift of Dr. A. Malik, Department of Pharmacology, The University of Illinois College of Medicine, Chicago, Illinois. All chemicals and reagents were purchased from Sigma unless otherwise specified.

### Leukostasis

Anesthetized mice were perfused at 200mmHg (Infusurge 4010 pressure infuser), 18mL/min via the aorta for 2 minutes with warm saline followed by 10mL of PBS containing 200uL of Concanavalin A FITC (Vector 5mg/mL). Residual Concanavalin A was washed away with an additional 2 min of perfusion with saline. The retina was isolated as detailed below and FITC-Concanavalin A-labeled leukocytes adherent to the walls of retinal blood vessels were counted as the number of observable leukocytes per retina using fluorescent microscopy [20,26,27].

### Leukocyte isolation

0.5-1mL of whole blood was drawn via cardiac puncture from CO_2_ anesthetized mice using a syringe containing 0.05mL of 7.2mg EDTA /mL PBS. Blood was immediately transferred to 7.2mg EDTA vaccutainer tubes (BD) and stored on ice for 2-10 minutes. Whole blood was incubated with 3 mL of RBC lysis buffer (Ebioscience) on ice for 5 minutes, centrifuged at 200xg for 7 minutes, re-suspended in 3 mL of lysis buffer, centrifuged again, and washed twice in Krebs-Hepes buffer (HBSS (Mediatech, 1/10 normal calcium), 0.02M Hepes (Gibco) and 5 or 25mM glucose for leukocytes from non-diabetic or diabetic donor mice, respectively) [13].

### Retina isolation

The anterior section was removed from enucleated eyes of perfused mice with Teflon coated razor blades (Electron Microscopy Sciences). Intact retina was carefully separated from any attachment at the ciliary body and sclera with a micro-spatula, and at the optic nerve with micro dissection scissors. Incisions in a clover leaf pattern were made to ease mounting on microscope slides and prevent folding during the superoxide assay.

### Superoxide

Retinae were incubated in 200uL Krebs-Hepes buffer with 5 or 25 mM glucose for 5 min, at 37°C, 5% CO2. Leukocytes (400,000 cells per 400uL buffer) were incubated under identical conditions for 25 minutes. Luminescence indicating the presence of superoxide was measured 5 minutes after the addition of 0.54mM (final concentration) Lucigenin using a luminometer (Monolight 2010) [28,29]. 

### Histopathology

Eyes enucleated from mice diabetic for 40 weeks and age-matched non-diabetic controls were fixed in buffered formalin for at least one week. The fixed retina was isolated, rinsed in running water overnight, and then digested with 40 U/mL elastase (Calbiochem), 5mM EDTA 100mM sodium phosphate, and 150mM NaCl pH6.5 at 37°C for 2-3 hours [30]. Nonvascular cells were brushed away over the ensuing day, and the isolated vascular beds were transferred to charged microscope slides (Superfrost/Plus Fisher Scientific) and allowed to dry prior to PASH (periodic acid, schiff base, hematoxylin) staining. 

The number of capillary junctions (an estimate of the number of capillaries) and nuclei of blood vessels (both pericyte and endothelium) per square mm were counted in non-diabetic WT and NIF^+/-^ expressing mice to determine if the presence of NIF altered normal vascular development in the retina.

Degenerate (acellular) capillaries were quantified in 6-8 fields in the mid-retina across all quadrants of retina as previously reported [4,31]. Briefly acellular capillaries were counted in 0.419 mm^2^ fields centered halfway between the optic nerve and the periphery. Capillaries were considered acellular only if no nuclei were in present in the capillary prior to the capillary junction. Capillaries were counted only if the width of the capillary at any point prior to a junction was at least 20% of normal capillary width and if the capillary length was at least three times normal capillary width. 

### Co-Culture

A mouse retinal endothelial cell line (mREC; generated from Immortomice) [32], greater than 99% positive for CD31 by flow cytometry, was cultured on 10 cm gelatin-coated plates in DMEM with 10% FBS and 5.5 or 25mM glucose, which was changed every other day until the cells were 80% confluent. Leukocytes (200,000/ plate) purified from whole blood were added for 24 hours, after which mREC were gently rinsed with PBS to remove leukocytes, incubated with trypsin for 2 minutes, and washed twice in PBS. Viability of mREC was measured by trypan blue exclusion with a hemocytometer [13]. Sample identity was masked during counting. mREC [32] were the generous gift of Dr. Nader Sheibani, Department of Ophthalmology and Visual Sciences, University of Wisconsin, Madison, Wisconsin, United States of America

### Bacterial clearance in air pouch model

A skin abscess air pouch model [33,34] was used to assess the ability of mice to clear a bacterial infection in the presence of NIF. Briefly, an air pouch was created on day 1 by injecting anesthetized mice with 5 mL of sterile air under the skin above the spine. On day 4 the air pouch was re-inflated with 3mL of air. On day 7, Luria broth (LB) was inoculated with *Klebsiella pneumoniae* (ST258 UHKPC SS7 N1181172 677/1607) from an overnight culture at RT, which was then grown to 0.6 OD at 37°C. Bacteria were washed 3 times in PBS (500xg, 10 minutes) and diluted to 0.2 OD. 300x10^6^ bacteria in PBS (=0.5mL of 0.2OD bacteria) were injected into the air pouch. Twenty-four hours later, the air pouch of anesthetized mice was lavaged with 3mL of Hank’s Balanced Salt Solution (HBSS, Mediatech) containing 0.15 M sodium citrate, and a hemocytometer was used to count the number of leukocytes present in the lavage fluid. 200uL of the lavage fluid was immediately mixed with 2uL of 10% Triton-x 100 to lyse leukocytes containing live bacteria, and stored on wet ice until after leukocytes were counted in the remaining lavage fluid. 1/100 serial dilutions of the lysed solution were plated onto LB agar. Colony forming units (CFU) on the LB plates were counted the next day after overnight growth at 37°C. Dilutions of lavage fluid which resulted in greater than 200 CFU per plate were regarded as too numerous to count reliably. In such cases the next serial dilution of plated lavage fluid was used to calculate the number of CFU in the undiluted lavage fluid. Klebsiella pneumonia was the generous gift of Dr. R. Bonomo, Louis Stokes Veterans Affairs Medical Center, Cleveland, Ohio, United States of America.

### Statistics

Groups were compared using ANOVA followed by Fisher post-hoc test to generate p values. Error bars in graphs represent±1 SD. Sample sizes are indicated in the figure legends or on the figure.

## Results

NIF is a hookworm derived protein that binds to integrin CD11b/CD18. The effect of NIF on the development of retinal lesions (capillary loss and leukostasis) and pathogenic markers (superoxide production) characteristic of the early stages of diabetic retinopathy were measured in experimentally diabetic mice and non-diabetic controls.

### Effect of NIF on normal blood vessel development in the retina

**Figure 1:**
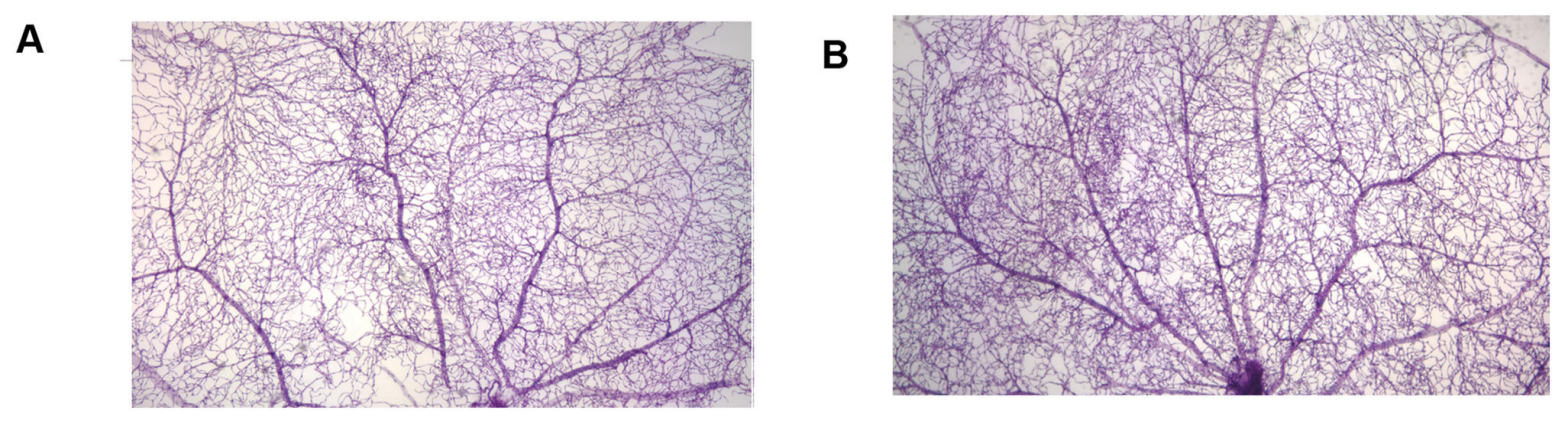
Neutrophil inhibitory factor (NIF) does not inhibit normal vascular development in the retina. No differences in capillary density were apparent in elastase digested vascular preparations of fixed retina isolated from A) non-diabetic WT, B) non-diabetic NIF^+/-^ mice. Images were captured at 40x.

No significance differences (p>0.05, n=6) were found between the number of capillaries (junctions) 812±78/ mm^2^ (WT) and 841±68/ mm^2^ (NIF^+/-^) or the number of nucleated cells in blood vessels 2510±247/ mm^2^ (WT) and 2494±245/ mm^2^ (NIF+/-). In addition, no gross morphological changes were apparent in the isolated retinal vasculature (Figure 1).

### Evaluation of diabetes

Experimental induction of diabetes with streptozotocin (SD) resulted in a significant increase of non-fasted blood glucose (p<0.001) and glycated hemoglobin HbA1c (p<0.001) in diabetic WT mice compared to non-diabetic WT controls over the 40 weeks of study (Table 1). Data at 10 weeks diabetes was similar. The presence of NIF in non-diabetic or diabetic animals did not significantly alter blood glucose values or HbA1c. 

**Table 1:** Evaluation of diabetes.

Group	Streptozotocin	Duration	non-fasted blood glucose	H1Ac	Weight	n
	Diabetic	(weeks)	(mg/dL)	(%)	(g)	
WT	no	32	114±16	4.0±0.9	62±9	10
NIF^+^	no	32	137±27	3.9±0.2	66±16	10
WT	diabetic	32	410±73	9.3±1.1	72±10	9
NIF^+^	diabetic	32	451±74	9.6±0.9	70±11	10
WT	no	10	167±22	3.1±0.1	59±5	10
NIF^+^	no	10	162±18	3.2±0.1	58±12	10
WT	diabetic	10	475±60	8.4±0.5	46±3	10
NIF^+^	diabetic	10	488±58	8.4±0.4	44±8	12

p values are listed in the results section

### Capillary loss in the retina

**Figure 2:**
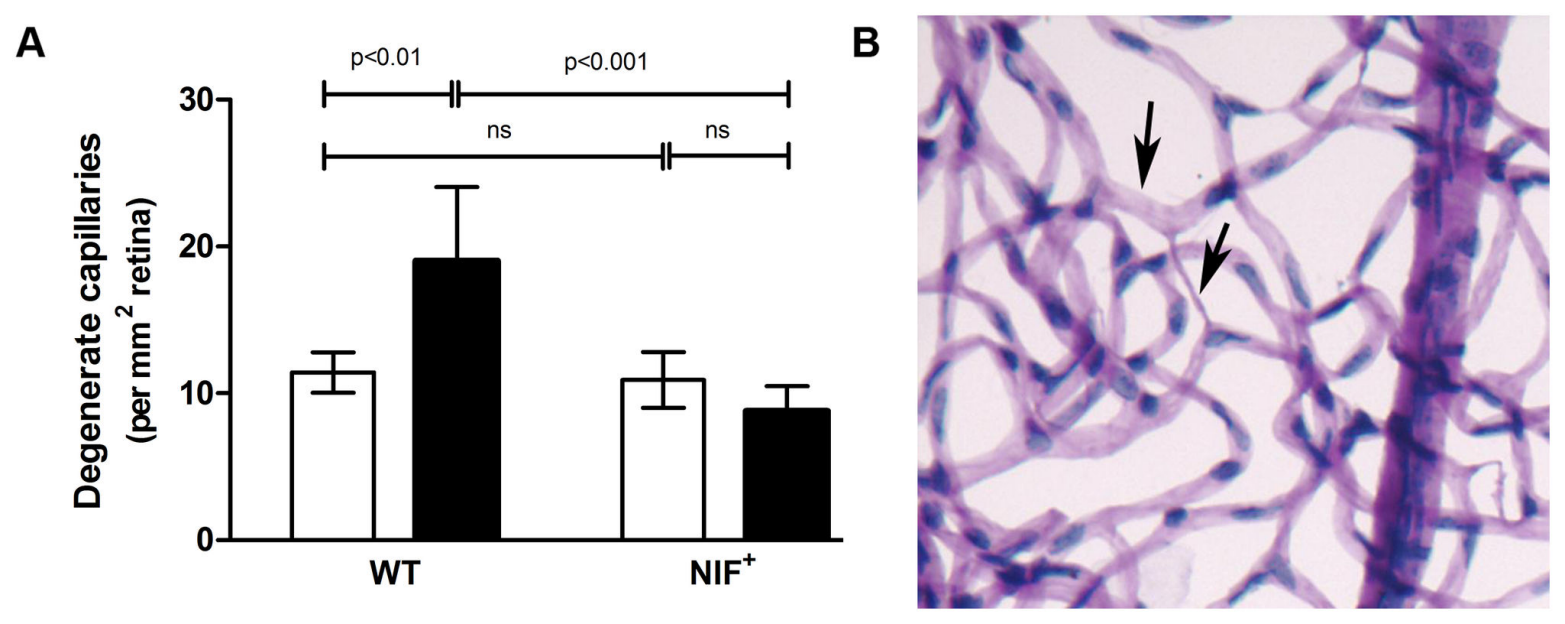
NIF inhibits diabetes-induced retinal capillary loss. A) WT mice diabetic for 40 weeks developed significantly more retinal capillary loss than non-diabetic controls. Mice expressing NIF did not develop the diabetes-induced retinal capillary loss and were not significantly different from non-diabetic controls. Streptozotocin induced diabetic mice (SD) are black bars and non-diabetic (ND) mice are white bars (n=4 for non-diabetic and diabetic WT animals, n= 6 for non-diabetic NIF expressing animals, and n= 5 for diabetic NIF expressing animals). B) Retinal vasculature isolated from diabetic WT mouse. Black arrows indicate acellular capillaries.

Diabetes-induced retinal vascular capillary loss is an important clinical endpoint in the development and progression of DR. Degeneration of retinal capillaries in WT mice diabetic for 40 weeks was significantly greater than in age-matched non-diabetic WT mice (Figure 2). In contrast, diabetic mice expressing NIF had significantly less capillary loss than did WT diabetic controls. Thus, diabetes-induced retinal capillary loss can be inhibited by selective α_m_β_2_ leukocyte integrin antagonism.

### Leukostasis

**Figure 3:**
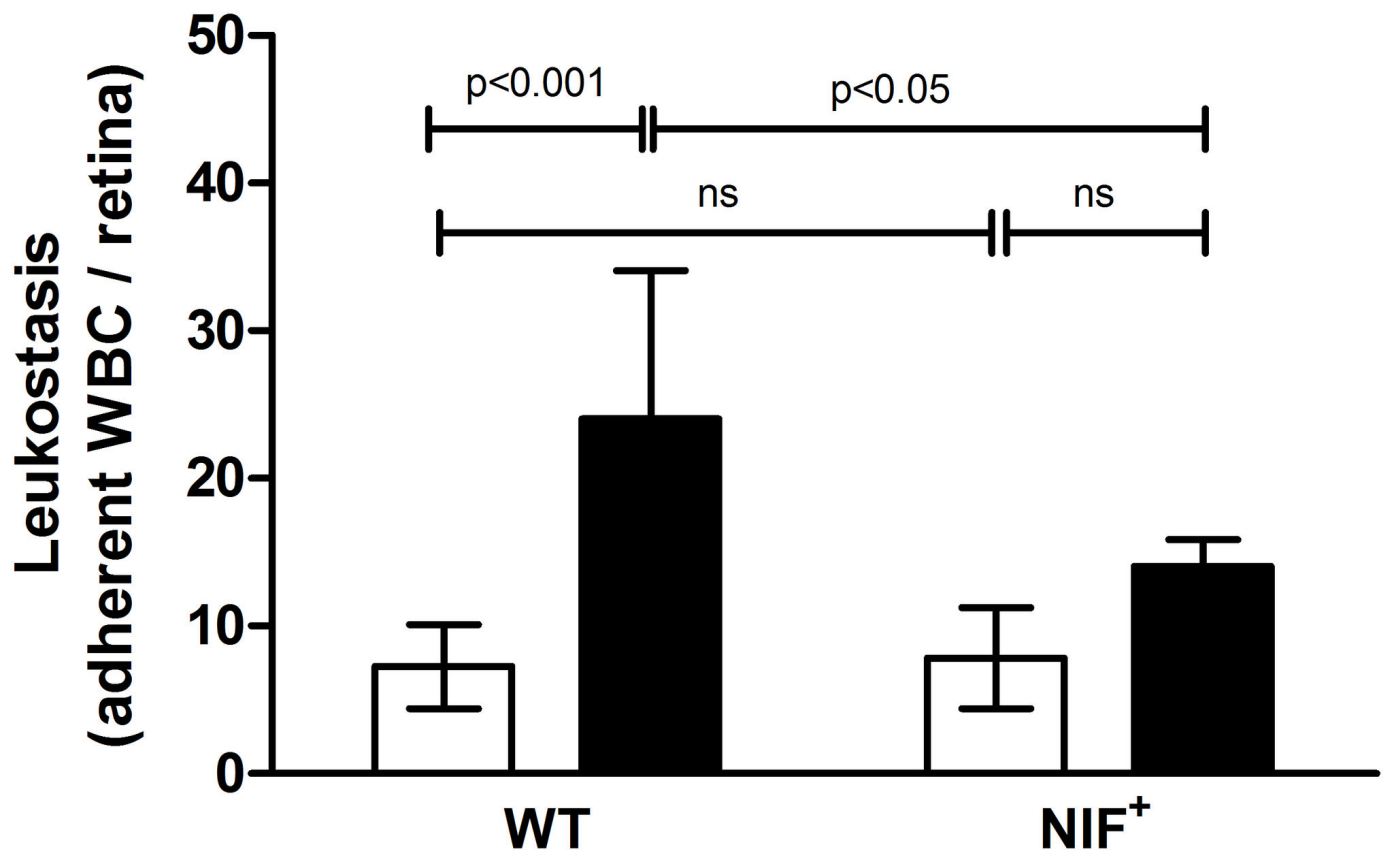
NIF significantly decreases diabetes-induced leukostasis in the retinal vasculature. Diabetes significantly increased the number of adherent leukocytes in the retinal vasculature in WT mice but not in diabetics expressing NIF. Duration of diabetes was 10 weeks. Streptozotocin induced diabetic mice (SD) are black bars and non-diabetic (ND) mice are white bars (n=5).

The number of leukocytes adherent to the vascular endothelium of the retina are observed to increase in diabetic mice [8] and humans [9]. The adherent leukocytes have been postulated to contribute to occlusion and degeneration of retinal blood vessels in diabetes [11]. Ten weeks after the onset of diabetes, WT mice showed the expected diabetes-induced significant increase in leukostasis in the retina (Figure 3), whereas diabetic mice expressing NIF did not develop a significant increase in retinal leukostasis. Since NIF is a selective antagonist for α_m_β_2_, the results suggest that diabetes-induced leukostasis in the retina is largely due to leukocyte α_m_β_2_ binding to a ligand on the endothelial cell. 

### Retinal and leukocyte superoxide

**Figure 4:**
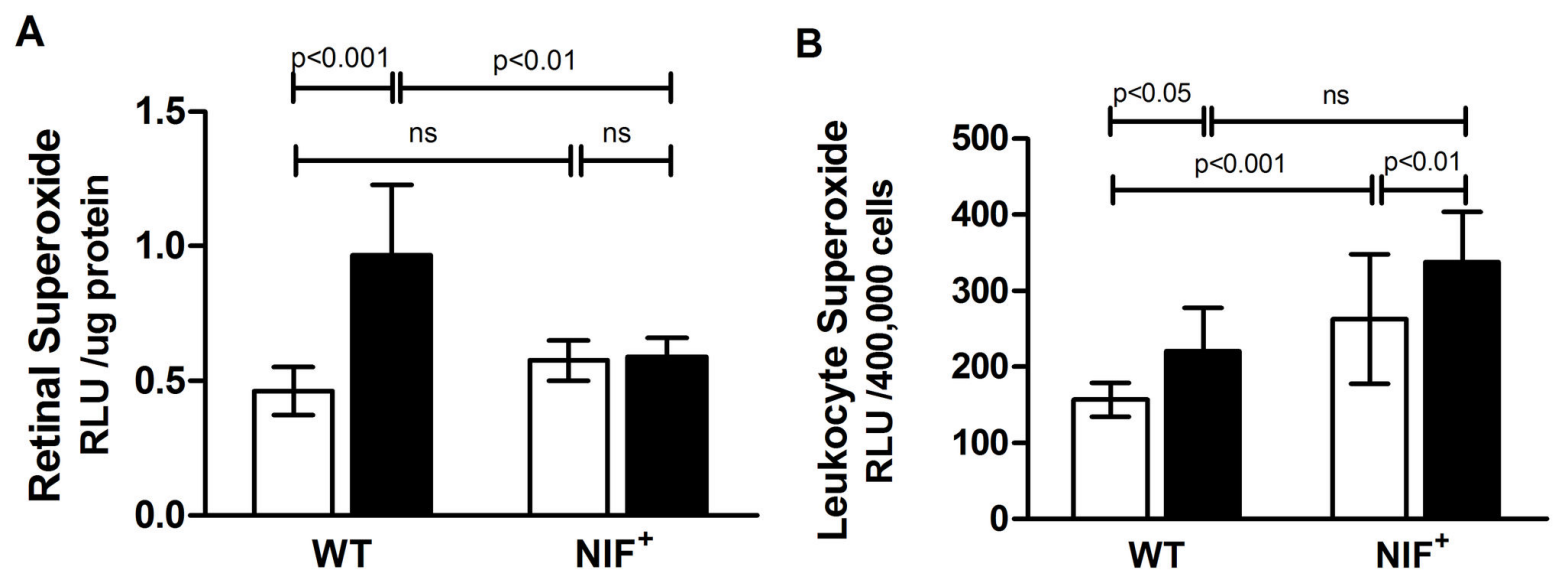
NIF inhibits the diabetes-induced up-regulation of superoxide in retina but not circulating leukocytes. A) Superoxide generation increased in the retina of WT animals diabetic for 10 weeks, but not in diabetics expressing NIF (n=5). B) Superoxide production is increased also in leukocytes of WT animals diabetic 10 weeks compared to non-diabetic controls. In contrast, the presence of NIF resulted in an elevated leukocyte superoxide production in non-diabetic animals which was further increased in NIF mice diabetic for 10 weeks (n=5).

Superoxide production is increased in the retina of diabetic mice and has been implicated in the pathogenesis of DR. By 10 weeks of diabetes, WT mice developed a significant increase in retinal superoxide production. In contrast, diabetic mice expressing NIF did not produce greater than normal amounts of superoxide. (Figure 4A) 

Leukocyte superoxide production has been previously reported to be increased in mice after several weeks of diabetes, suggesting that the leukocytes are activated in diabetic animals [13]. Consistent with this, WT mice developed a significant increase in leukocyte superoxide production by 10 weeks after the onset of diabetes. (Figure 4B) Interestingly, the leukocyte generation of superoxide was not inhibited in mice expressing NIF, and in fact was significantly increased in non-diabetic mice expressing NIF compared to WT controls. Leukocyte superoxide production in diabetic mice expressing NIF appeared to increase but was not significantly different from diabetic WT controls.

### Leukocyte-mediated killing of endothelial cells

**Figure 5:**
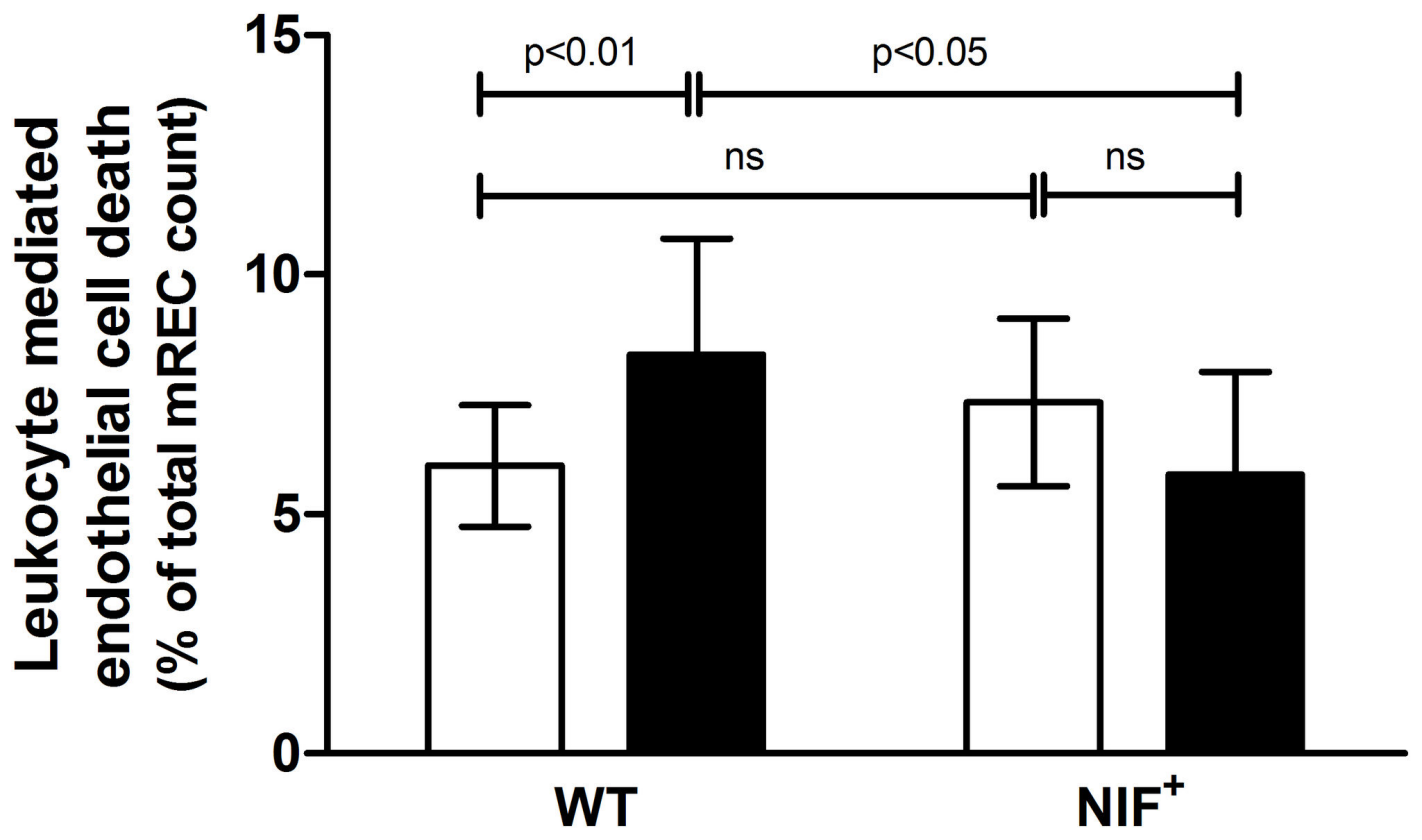
NIF inhibits endothelial cell death caused by leukocytes isolated from diabetic animal. Co-culture of transformed mouse retinal endothelial cells (mREC) with leukocytes resulted in significant increases in the number of dead endothelial cells when leukocytes were isolated from WT diabetic animals compared to leukocytes harvested from WT non-diabetic animals. In contrast, leukocytes isolated from diabetic animals expressing NIF did not significantly increase the number of endothelial cells killed in the co-culture assay compared to non-diabetic animals expressing NIF or diabetic WT animals (n=3).

The ability of leukocytes from diabetic animals to induce capillary death was investigated in vitro using co-cultures of endothelial cells and fresh leukocytes. Leukocytes added to plates of mouse retinal endothelial cells in vitro were observed to settle onto the endothelial cells within minutes. Overnight incubation of the endothelial cells with leukocytes from WT diabetic mice resulted in significant increases in the number of dead endothelial cells compared to the number of dead endothelial cells observed when using leukocytes from non-diabetic controls. (Figure 5) In contrast, leukocytes harvested from either diabetic or non-diabetic animals expressing NIF did not significantly elevate the number of dead endothelial cells. We conclude that NIF inhibits the ability of leukocytes activated by diabetes to kill endothelial cells.

### Bacterial Clearance

NIF interferes with leukocyte binding and function, and therefore might decrease the ability of the immune system to respond to microbial infections. Mice expressing NIF did not exhibit any increase in incidence of sickness (general observation of activity and kyphosis) or morbidity compared to WT controls, suggesting that their immune system was not critically impaired (data not shown). A skin pouch abscess model with an opportunistic strain of bacteria was used to more rigorously evaluate the ability of leukocytes to migrate to, and eliminate, a bacterial challenge in control and NIF-expressing mice. 

**Figure 6:**
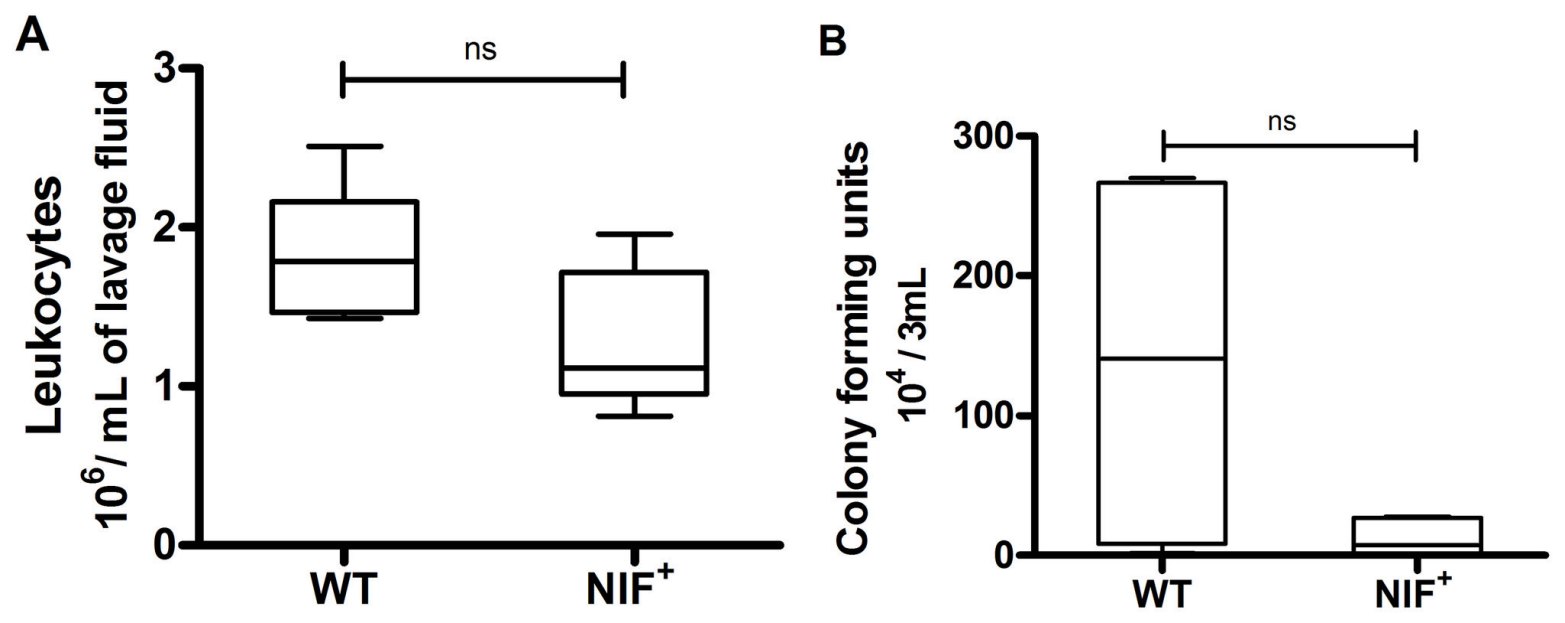
NIF does not significantly inhibit immune response to microbial challenge. *K.Pneumonia* injected into an air pouch abscess model elicited leukocyte migration into the pouch. A) The number of leukocytes recovered in 3 mL of lavage fluid 24 hours after bacterial challenge was not significantly different between mice expressing NIF and WT controls. The presence of NIF did not inhibit leukocyte migration (and required chemotaxis) into the site of the inoculation (n=5). B) The number of colony forming units, representing residual living *K.Pneumonia* in the lavage fluid, recovered 24 hours after bacterial challenge, was not significantly increased in the presence of NIF. Therefore the presence of NIF did not inhibit the ability of leukocytes to phagocytize and kill *K.Pneumonia* (n= 4).

The number of leukocytes present in lavage fluid harvested 24 hours after inoculation with bacteria was not significantly reduced in animals expressing NIF compared to WT controls. (Figure 6A) Thus, NIF did not significantly impair the ability of the host to recruit leukocytes to the site of infection (including chemokine production, leukocyte chemotaxis, or leukocyte diapedesis).

The number of colony forming units (live bacteria) recovered from the lavage fluid was not significantly increased when the lavage fluid was harvested from NIF expressing mice compared to WT controls (Figure 6B), indicating that NIF did not significantly impair the ability of the leukocytes to phagocytize or to kill the phagocytized bacteria. 

## Discussion

Several pieces of data indicate that leukocytes and leukocyte adhesion to endothelial cells play a critical role in diabetes-induced retinal capillary loss. First, deletion of proteins mediating leukocyte adhesion, ICAM1 or its binding partner on leukocytes integrin subunit β2 (CD18), significantly inhibited the diabetes-induced vascular permeability and degeneration of retinal capillaries in animals [35]. Furthermore, diabetes-induced retinal capillary loss was also inhibited in animals deficient in leukocyte and inflammation associated proteins such as iNOS or PARP1 [26]. Adoptive transplantation of leukocytes (bone-marrow) deficient in iNOS or PARP1 was sufficient to inhibit diabetes-induced retinal capillary degeneration in WT animals. In the reverse experiment, adoptive transfer of WT leukocytes (marrow) into animals deficient in iNOS was sufficient to restore the diabetes-induced retinal capillary degeneration demonstrating that leukocytes were required to develop the diabetes-induced retinal vascular pathology [13]. In addition, leukocyte-mediated endothelial injury has been shown in vitro to be greater when the leukocytes are isolated from diabetic mice [13] or patients [36] than when using leukocytes isolated from their non-diabetic counterparts. 

The current results provide evidence that NIF-mediated blockade of the leukocyte α_m_β_2_ integrin significantly inhibited a number of important diabetes-induced abnormalities in retina. Notable among these abnormalities inhibited by NIF is the important clinical endpoint of diabetes-induced degeneration of retinal capillaries. NIF also inhibited the diabetes-induced increase in leukocytes adhering to retinal blood vessels. Since NIF binds to, and blocks, the α_m_β_2_ integrin, this was not unexpected, but it does demonstrate a long-term effect of NIF, for which compensation (adhesion through LFA-1 binding) did not occur. NIF protein expression patterns and serum concentration in NIF^+/-^ CD1 mice could not be independently confirmed due to the current lack of an available anti-NIF antibody, however NIF has been reported to be found in all organs of NIF^+/+^ mice[25].

Mice expressing NIF (NIF^+/+^ CD1) have been reported to have increased circulating neutrophils and decreased lymphocytes compared to non-NIF expressing CD1 mice [25] and a shift in the sub populations of leukocytes might be a factor in the observed differences in diabetes-induced retinal lesions. This seems unlikely to explain our results, however, as we have previously reported that neutrophils were a major cause of the retinopathy in diabetic mice [13], and therefore an increase in neutrophils would be expected to cause more vascular pathology not less as was observed.

NIF also inhibited the diabetes-induced increase in retinal superoxide production. We previously have observed that the ability of a therapy or genetic manipulation to inhibit the diabetes-induced generation of superoxide by the retina strongly predicts the ability of that therapy to inhibit retinal capillary degeneration in long-term studies of diabetic rodents [27,28]. The inhibition of diabetes-induced retinal production of superoxide by blocking α_m_β_2_ integrin and associated leukocyte binding to endothelial cells in the present report is consistent with our previous report demonstrating that leukocytes were required for diabetes-induced generation of superoxide by the retina [13]. Our results suggest that retinal superoxide produced by an unidentified cell type in the retina is mediated by leukocyte α_m_β_2_ interaction with capillary endothelial cells.

Leukocytes from animals expressing NIF exhibited increased superoxide production compared to WT controls (Figure 4) however the increase in production was only significant in non-diabetic NIF expressing animals. The results suggest that NIF may bind as a partially activating ligand consistent with recent observations that integrin receptors can adopt multiple confirmations to yield different functions [37]. Despite the ability of NIF to inhibit superoxide generation by the retina, NIF did not inhibit diabetes-induced superoxide release by leukocytes, similar to the reported inability of NIF to inhibit PMA-activated leukocyte superoxide production [38]. 

Leukocytes isolated from diabetic or non-diabetic animals expressing NIF did not cause increased death of retinal endothelial cells, even though they generated more superoxide than did leukocytes from animals not expressing NIF. These results are consistent with previous studies demonstrating that anti-α_m_β_2_ antibody blocked endothelial cell injury from activated leukocytes without inhibiting superoxide production by those leukocytes [38,39]. We conclude that the superoxide released by leukocytes “activated” by diabetes or by NIF ligation to the α_m_β_2_ integrin is not sufficient to cause the diabetes-induced leukocyte mediated endothelial death.

Therapies targeting integrins other than MAC1, including humanized anti-CD11a, anti-ICAM1 and anti-CD18, have been associated with increased incidence of infection in clinical trials, including increased fungal and bacterial infections, [40–43]. Immune related complications in anti-integrin therapy are not unexpected since humans deficient in CD18 (leukocyte adhesion deficiency I) also are immunocompromised and prone to infections. Thus, inhibition of leukocyte-mediated damage to endothelial cells without simultaneously compromising the immune system requires careful selection of the target. In our hands, NIF blocked the diabetes-induced degeneration of retinal capillaries, yet did not impair immune function, as evidenced by near normal leukocyte recruitment and bacterial elimination when directly challenged with an opportunistic organism. The apparent decrease in residual bacteria in the NIF expressing animals might be related to the increase in superoxide generation by leukocytes in those animals. 

NIF has previously been administered to patients as an acute therapy after stroke without adverse effects including incidence of infection [44]. Although NIF demonstrated improvement in patient recovery following a stroke when administered with tissue plasminogen activator (tPA), NIF alone failed to demonstrate significant improvement. While it is not possible to discern the reason for the failure of the trial from the published data, the authors noted that patients in the study were not screened for thromboembolism (non-resolved clot) for which NIF was previously shown to be effective only when co-administered with tPA [45]. Subsequently, this therapeutic approach was abandoned. The present study demonstrating that NIF significantly inhibits development of a slowly-developing retinopathy in diabetes suggests that the careful inhibition of adhesion between activated leukocytes and the vascular wall is still a valid therapeutic target for diabetes and possibly a variety of other chronic inflammatory diseases. 

In contrast to our results demonstrating a beneficial effect of inhibiting the interaction between leukocytes and endothelial cells, other investigators have reported that enhancing the binding of leukocytes to endothelial cells inhibited vascular disease such as neointimal thickening following denudation [46]. However this approach may be less efficacious in diabetes where adhesion of activated leukocytes may cause additional occlusion of capillaries and leukocyte-mediated endothelial damage. 

Even though NIF had therapeutic value in the present study of diabetic retinopathy, it is unlikely that NIF in its current form will be suitable for chronic administration to inhibit diabetic complications. NIF is a foreign protein, and therefore may lead to generation of anti-NIF antibodies, as has been observed when NIF was used as a vaccine [47]. To develop a therapy for clinical use in chronic diseases will require development of a small molecule or peptide that reproduces beneficial actions of NIF without inducing an undesirable immunologic response.

We conclude that ligation of the α_m_β_2_ integrin on leukocytes with a molecule on the surface of endothelial cells (presumably ICAM-1) is critical to the diabetes-induced degeneration of retinal endothelial cells. NIF blocks this interaction, and therefore inhibits the diabetes-induced retinal capillary degeneration that is characteristic of early diabetic retinopathy. Importantly, NIF accomplishes this without inhibiting the normal immune response to infection.
